# Augmented Cardiac Growth Hormone Signaling Contributes to Cardiomyopathy Following Genetic Disruption of the Cardiomyocyte Circadian Clock

**DOI:** 10.3389/fphar.2022.836725

**Published:** 2022-02-16

**Authors:** Ravi Sonkar, Ryan Berry, Mary N. Latimer, Sumanth D. Prabhu, Martin E. Young, Stuart J. Frank

**Affiliations:** ^1^ Division of Endocrinology, Diabetes and Metabolism, Department of Medicine, University of Alabama at Birmingham, Birmingham, AL, United States; ^2^ Division of Cardiovascular Disease, Department of Medicine, University of Alabama at Birmingham, Birmingham, AL, United States; ^3^ Cardiology Section, Birmingham VAMC Medical Service, Birmingham, AL, United States; ^4^ Division of Cardiology, Washington University School of Medicine, St. Louis, MO, United States; ^5^ Endocrinology Section, Birmingham VAMC Medical Service, Birmingham, AL, United States

**Keywords:** chronobiology, fibrosis, heart, hypertrophy, signaling

## Abstract

Circadian clocks regulate numerous biological processes, at whole body, organ, and cellular levels. This includes both hormone secretion and target tissue sensitivity. Although growth hormone (GH) secretion is time-of-day-dependent (increased pulse amplitude during the sleep period), little is known regarding whether circadian clocks modulate GH sensitivity in target tissues. GH acts in part through induction of insulin-like growth factor 1 (IGF1), and excess GH/IGF1 signaling has been linked to pathologies such as insulin resistance, acromegaly, and cardiomyopathy. Interestingly, genetic disruption of the cardiomyocyte circadian clock leads to cardiac adverse remodeling, contractile dysfunction, and reduced lifespan. These observations led to the hypothesis that the cardiomyopathy observed following cardiomyocyte circadian clock disruption may be secondary to chronic activation of cardiac GH/IGF1 signaling. Here, we report that cardiomyocyte-specific BMAL1 knockout (CBK) mice exhibit increased cardiac GH sensitivity, as evidenced by augmented GH-induced STAT5 phosphorylation (relative to littermate controls) in the heart (but not in the liver). Moreover, *Igf1* mRNA levels are approximately 2-fold higher in CBK hearts (but not in livers), associated with markers of GH/IGF1 signaling activation (e.g., p-ERK, p-mTOR, and p-4EBP1) and adverse remodeling (e.g., cardiomyocyte hypertrophy and interstitial fibrosis). Genetic deletion of one allele of the GH receptor (GHR) normalized cardiac *Igf1* levels in CBK hearts, associated with a partial normalization of adverse remodeling. This included attenuated progression of cardiomyopathy in CBK mice. Collectively, these observations suggest that excessive cardiac GH/IGF1 signaling contributes toward cardiomyopathy following genetic disruption of the cardiomyocyte circadian clock.

## Introduction

Virtually all aspects of life fluctuate over the course of the day, often in parallel with environmental factors (such as light intensity and ambient temperature). In mammals, 24-h oscillations have been reported at cellular (*e.g.,* transcription, translation, signaling), organ (*e.g.,* muscle contractility, cognitive function), and whole body (*e.g.,* sleep-wake and fasting-feeding behaviors) levels (Takahashi et al., 2008). Such 24h oscillations in biological processes are not solely the consequence of daily changes in environmental factors, as multiple rhythms persist during constant exogenous conditions (Takahashi et al., 2008). These intrinsic 24 h oscillations are termed circadian rhythms, and are driven by internal circadian clocks. Circadian clocks are cell autonomous molecular mechanisms, consisting of transcriptional-translational feedback loops, with a periodicity of approximately 24 h (Edery, 2000). It has been estimated that mammalian circadian clocks regulate between 3 and 16% of an organ’s transcriptome, resulting in circadian governance of a diverse array of biological processes (*e.g.,* hormone secretion, metabolic flux, signal transduction, etc.) (Zhang et al., 2014). It has been hypothesized that temporal partitioning of cellular functions by the circadian clock confers the selective advantage of anticipation, allowing a cell to prepare for an extrinsic stimulus or stress prior to its onset (Edery, 2000). In doing so, mammalian circadian clocks change the sensitivity/responsiveness of cells/organs to various neurohumoral factors over the course of the day. The importance of circadian governance is underscored by reports that attenuation/manipulation of circadian clocks through genetic (e.g., single nucleotide polymorphisms in humans or gene ablation in animal models) and/or environmental (e.g., shift work in humans or light/dark manipulation in animal models) means invariably increases pathology risk (e.g., obesity, diabetes mellitus, cardiovascular disease) (Knutsson et al., 1986; Turek et al., 2005; Davidson et al., 2006; Woon et al., 2007; Scott et al., 2008; Marcheva et al., 2010; Lefta et al., 2012; Knutsson and Kempe, 2014).

Numerous cardiovascular parameters are under circadian influence. Heart rate and blood pressure both fluctuate over a 24 h period (Degaute et al., 1991). Human studies designed to eliminate environmental and/or behavioral influences highlight circadian governance of key cardiovascular parameters (Scheer et al., 2010; Shea et al., 2011). Moreover, 24 h rhythms in heart rate are observed in *ex vivo* perfused hearts and isolated cardiomyocytes, consistent with mediation by an intrinsic timekeeping mechanism (Bray et al., 2008; D'Souza et al., 2020; du Pré et al., 2017). Circadian clocks are composed of a series of transcriptional modulators, of which CLOCK (circadian locomotor output cycles kaput) and BMAL1 (brain and muscle ARNT-like 1) play a central role; genetic manipulation of CLOCK and/or BMAL1 often disrupts the circadian clock mechanism (Vitaterna et al., 1994; Hogenesch et al., 1998). Through the use of dominant negative CLOCK mutant and BMAL1 knockout mice (both germline and cell type specific), circadian clocks have been reported to regulate not only heart rate and blood pressure, but also cardiac metabolism, signaling, and electrophysiology (Curtis et al., 2007; Bray et al., 2008; Schroder et al., 2013; Young et al., 2014; Schroder et al., 2015; McGinnis et al., 2017). Moreover, the cardiomyocyte circadian clock modulates responsiveness of the heart to both physiologic stimuli (*e.g.,* insulin, adrenergic agonists, fatty acids) and pathologic stress (*e.g.,* prohypertrophic stimuli, ischemia/reperfusion) (Durgan et al., 2006; Bray et al., 2008; Durgan et al., 2010; Durgan et al., 2011a; McGinnis et al., 2017). Highlighting the critical nature of this mechanism in the maintenance of cardiac function, both germline and cardiomyocyte-specific BMAL1 mouse models develop an age-onset dilated cardiomyopathy associated with reduced lifespan (Lefta et al., 2012; Young et al., 2014). However, the precise mechanisms by which circadian disruption leads to cardiomyopathy are currently unknown.

As with many endocrine factors, secretion of growth hormone (GH) varies as a function of time-of-day (with increased pulse amplitude during the sleep period) (Goji, 1993; Jaffe et al., 1998; Avram et al., 2005; Gamble et al., 2014). Upon binding to cell surface receptors, GH activates a signaling cascade in target tissues, ultimately resulting in insulin-like growth factor 1 (IGF1) induction (Argetsinger et al., 1993; Frank and Messina, 2002). IGF1 subsequently acts in autocrine, paracrine, and endocrine manners (Favelyukis et al., 2001). Collectively, GH and IGF1 signaling impact numerous biological processes, including cellular growth and metabolism (Woelfle et al., 2005; Møller and Jørgensen, 2009). Chronic activation of this signaling system results in numerous pathologies, characterized by insulin resistance, organomegaly and cardiomyopathy (Clayton, 2003; Melmed, 2009; Arcopinto et al., 2013). The latter typically involves hypertrophic growth of cardiomyocytes, ventricular wall thickening, and chamber dilation (Braunwald, 2017). Indeed, cardiac IGF1 overexpression leads to adverse cardiac remodeling and contractile dysfunction (Delaughter et al., 1999). Interestingly, circulating IGF1 levels are chronically decreased following whole body deletion of cryptochromes (a core circadian clock component integral to one of the negative feedback loops) (Chaudhari et al., 2017). However, whether circadian disruption alters GH/IGF1 signaling in the cardiovascular system is currently unknown.

The purpose of the present study was to investigate whether altered GH/IGF1 signaling in the heart contributes towards adverse cardiac remodeling following disruption of the cardiomyocyte circadian clock. Here, we initially confirmed that cardiomyocyte-specific BMAL1 knockout (CBK) mice exhibit adverse cardiac remodeling, which is associated with increased GH sensitivity and activation of the GH/IGF1 signaling pathway. To investigate causality between augmented GH/IGF1 signaling and adverse cardiac remodeling, GHR haploinsufficiency was established in CBK mice. GHR haploinsufficiency normalized IGF1 levels in CBK hearts, which was associated with attenuation of cardiomyocyte hypertrophy, cardiac fibrosis, and contractile dysfunction. Collectively, these findings suggest that augmented cardiac GH/IGF1 signaling following genetic disruption of the cardiomyocyte circadian clock likely contributes towards cardiomyopathy development.

## Methods

### Animal Models

CBK (BMAL1^flox/flox^/α-MHC-CRE^+/−^) and littermate control (BMAL1^flox/flox^/α-MHC-CRE^−/−^; CBK Control) mice on the C57Bl/6J background were developed as described previously (Young et al., 2014). CBK mice were backcrossed with GHR-floxed mice (kind gift from Dr. John Kopchick at Ohio University) for generation of CBK mice with GHR haploinsufficiency (BMAL1^flox/flox^/α-MHC-CRE^+/−^/GHR^flox/WT^; CBKG) and littermate controls (BMAL1^flox/flox^/α-MHC-CRE^−/−^/GHR^flox/WT^; CBKG Control). All experimental mice were male and were housed by the Animal Resources Program at the University of Alabama at Birmingham (UAB), under temperature- (72 ± 2°F) and light- (325-lux; lights on period) controlled conditions. A strict 12-h light/12-h dark cycle regime was enforced (lights on at 6AM; zeitgeber time [ZT] 0); the light/dark cycle was maintained throughout these studies, facilitating investigation of diurnal variations (as opposed to circadian rhythms). Standard rodent chow was provided in an *ad libitum* fashion, unless otherwise stated. All animal procedures were conducted according to the “Guide for the Care and Use of Laboratory Animals” and were approved by the Institutional Animal Care and Use Committees at UAB.

### Growth Hormone Challenge

*In vivo* GH stimulation was performed as described previously (Berry et al., 2018). Briefly, 15 week old mice were individually housed in standard conditions. After a 1 week acclimatization period, mice were placed in wire bottom cages without food. Following a 6 h fast, mice were anesthetized, an abdominal incision was made, followed by injection of either saline (vehicle control) or human recombinant GH (50 μg/kg body weight; gift from Eli Lilly Co., Indianapolis, IN) into the inferior vena cava. Five minutes after saline or GH administration, heart and liver were excised rapidly and flash frozen in liquid nitrogen prior to biochemical analysis. Exogenous GH challenge was performed at the end of the light phase (i.e., ZT12), a time at which endogenous murine GH levels are typically low (Chang et al., 2017).

### Histologic Assessment

Cross sections from the middle region of the left ventricle were taken immediately upon removal of the heart, and were fixed in formalin for 24 h (followed by storage in 70% ethanol at 4°C prior to embedding and sectioning). Wheat germ agglutinin (WGA) staining was utilized for measurement of myocyte cross-sectional area; at least 45 myocytes were assessed per heart using ImageJ software (NIH), as described previously (Ingle et al., 2015). Picrosirius Red staining of collagen fibers was utilized for semi-quantitative measurement of left ventricular interstitial fibrosis, using ImageJ software (NIH), as described previously (Durgan et al., 2011a). Hearts were isolated from mice at ZT20 for histologic assessments, for consistency with RT-PCR assessments.

### Quantitative RT-PCR

RNA was extracted from hearts using standard procedures. Candidate gene expression analysis was performed by quantitative RT-PCR, using previously described methods (Gibson et al., 1996; Heid et al., 1996). For quantitative RT-PCR, either ThermoFisher (Mn00802831_m1, Mm00850544_g1, and Mm01208489_m1 for *Igf1*, *Jak2*, and *Socs2,* respectively) or custom-designed (*Bmal1* and *Ghr*) Taqman assay were utilized. For the *Bmal1* assay, sequences were as follows: forward primer, 5′-CAT​TGA​TGA​ATT​GGC​TTC​TTT​GG-3′; reverse primer, 5′-TCC​TTA​GCA​CGG​TGA​GTT​TAT​CTA​AC-3′; Taqman probe, 5′-TCC​TGG​ACA​TTG​CAT​TGC​ATG​TTG-3′. For the *Ghr* assay, sequences were as follows: forward primer, 5′-CAG​TTC​CAA​AGA​TTA​AAG​GGA​TTG​A-3′; reverse primer, 5′-TTA​TCA​TGA​ATG​CCT​AAG​ATG​GTG​TT-3′; Taqman probe, 5′-ACC​TCC​TCC​AAC​TTC​CCT​CCC-3′. All quantitative RT-PCR data are presented as fold change from an indicated control group. Hearts were isolated from mice at ZT20 for gene expression assessments, a time of day at which Bmal1 levels are elevated in control hearts (Young et al., 2014; Mia et al., 2020).

### Western Blotting

Qualitative analysis of protein expression and phosphorylation status was performed *via* standard western blotting procedures, as described previously (Durgan et al., 2011b). Briefly, 20–30 µg protein lysate was separated on polyacrylamide gels and transferred to PVDF membranes. Membranes were probed for the following targets: p-STAT5^Tyr694^ (Cell Signaling, 9351L), p-ERK 1/2^Thr202/Tyr204^ (Cell Signaling 9101), p-mTOR^Ser−2448^ (Cell Signaling, 2974), and p-4EBP1^Thr−37/46^ (Cell Signaling, 9459). Rabbit HRP-conjugated secondary antibodies (Cell Signaling 7076) were used for chemiluminescent detection with Luminata Forte Western Blotting substrate (Millipore, WBLUF0100). Densitometry data were normalized to total STAT5 (Santa Cruz Biotechnology, sc-835) or amido black. Importantly, in order to minimize the contribution that position on the gel might have on outcomes, samples were randomized on gels; samples were re-ordered post-imaging, only for the sake of illustration of representative images (note, all bands for representative images for an individual experiment were from the same gel; see Supplementary Figures S4, S5).

### Echocardiography

Cardiac function was assessed by echocardiography using a VisualSonics VeVo 3100 Imaging System (VisualSonics, Toronto, Canada), and was analyzed by VisualSonics software. Briefly, mice were anesthetized with 1.5–2% isoflurane in an oxygen mix; heart rate, respiratory rate, and body temperature (35–37°C) were monitored continuously throughout the procedure to ensure adequate depth of anesthesia.

### Statistical Analysis

Statistical analyses were performed using t-tests, two-way ANOVAs, or repeated measures of ANOVA (where applicable). Briefly, analyses were performed using Prism statistical software to investigate main effects of GH, genotype, and/or age, followed by Sidak post hoc analyses for pair-wise comparisons (indicated in Figures). In all analyses, the null hypothesis of no model effects was rejected at *p* < 0.05.

## Results

### Disruption of the Cardiomyocyte Circadian Clock Induces Cardiac Hypertrophy

**FIGURE 1:**
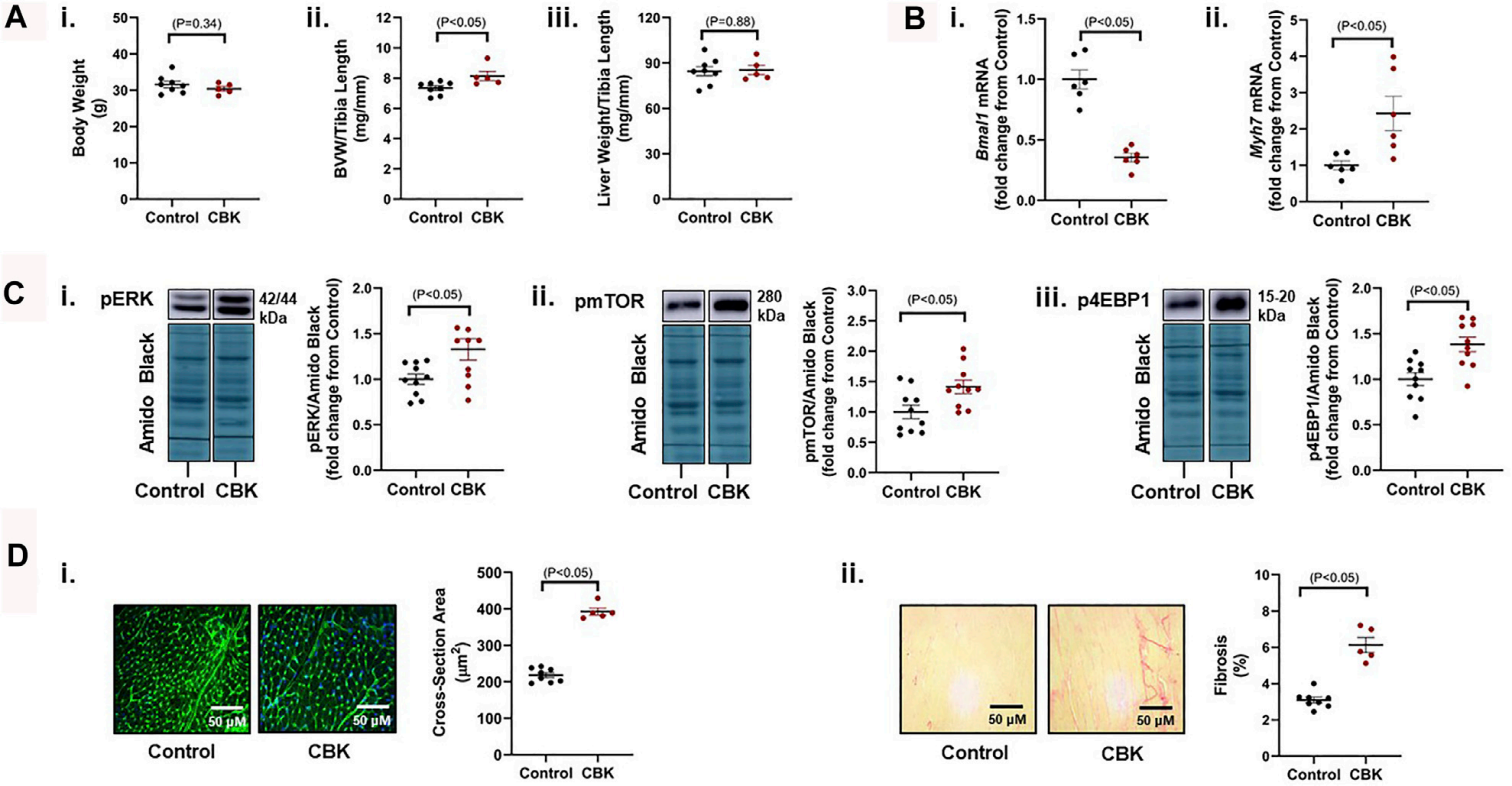
Phenotypic differences between 16 week old CBK and littermate control mice at the levels of gravimetric, gene expression, signaling, and histologic levels. **(A)** Body weight (i), biventricular weight to tibia length ratio (BVW/TL; ii), and liver weight to tibia length ratio (iii) (*n* = 5–8). **(B)** Cardiac *Bmal1* (i) and *Myh7* (ii) mRNA levels (*n* = 6). **(C)** p-ERK 1/2^Thr202/Tyr204^ (i), p-mTOR^Ser−2448^ (ii), and p-4EBP1^Thr−37/46^ (iii) protein levels (*n* = 10). **(D)** Cardiomyocyte size (i) and interstitial fibrosis (ii) (*n* = 5–8). Data/samples were collected at ZT20. All data are reported as mean ± SEM.

Genetic disruption of the circadian clock mechanism (both germline and cardiomyocyte-specific) results in adverse cardiac remodeling and dilated cardiomyopathy (Lefta et al., 2012; Kohsaka et al., 2014; Young et al., 2014; Ingle et al., 2015). This is exemplified by cardiomyocyte-specific BMAL1 knockout (CBK) mice, which have been reported to exhibit a hypertrophic phenotype by 12–16 weeks of age (Ingle et al., 2015). Here, we confirm that 16 week old CBK mice exhibit increased biventricular weight (BVW), in the absence of alterations in either body or liver weight (Figure 1A). As anticipated, cardiac *Bmal1* mRNA levels are decreased in CBK mice, associated with increased expression of the hypertrophic marker *Myh7* (Figure 1B); based on prior studies (Young et al., 2014), residual *Bmal1* mRNA in CBK hearts is secondary to contribution of non-cardiomyocyte cell types. Investigation of signaling components known to promote cardiac hypertrophy revealed increased phosphorylation of ERK, mTOR, and 4-EBP1 in CBK hearts (relative to littermate controls; Figure 1C); prior studies indicate that increased phosphorylation of these proteins are independent of absolute protein expression (McGinnis et al., 2017; Latimer et al., 2021). Histologic analysis confirmed increased cardiomyocyte cross-sectional area, as well as interstitial fibrosis, in CBK hearts (relative to littermate controls; Figure 1D). Collectively, these observations confirm a hypertrophic phenotype following genetic disruption of the cardiomyocyte circadian clock.

### Cardiomyocyte Circadian Clock Disruption Increases Cardiac Growth Hormone Sensitivity

**FIGURE 2:**
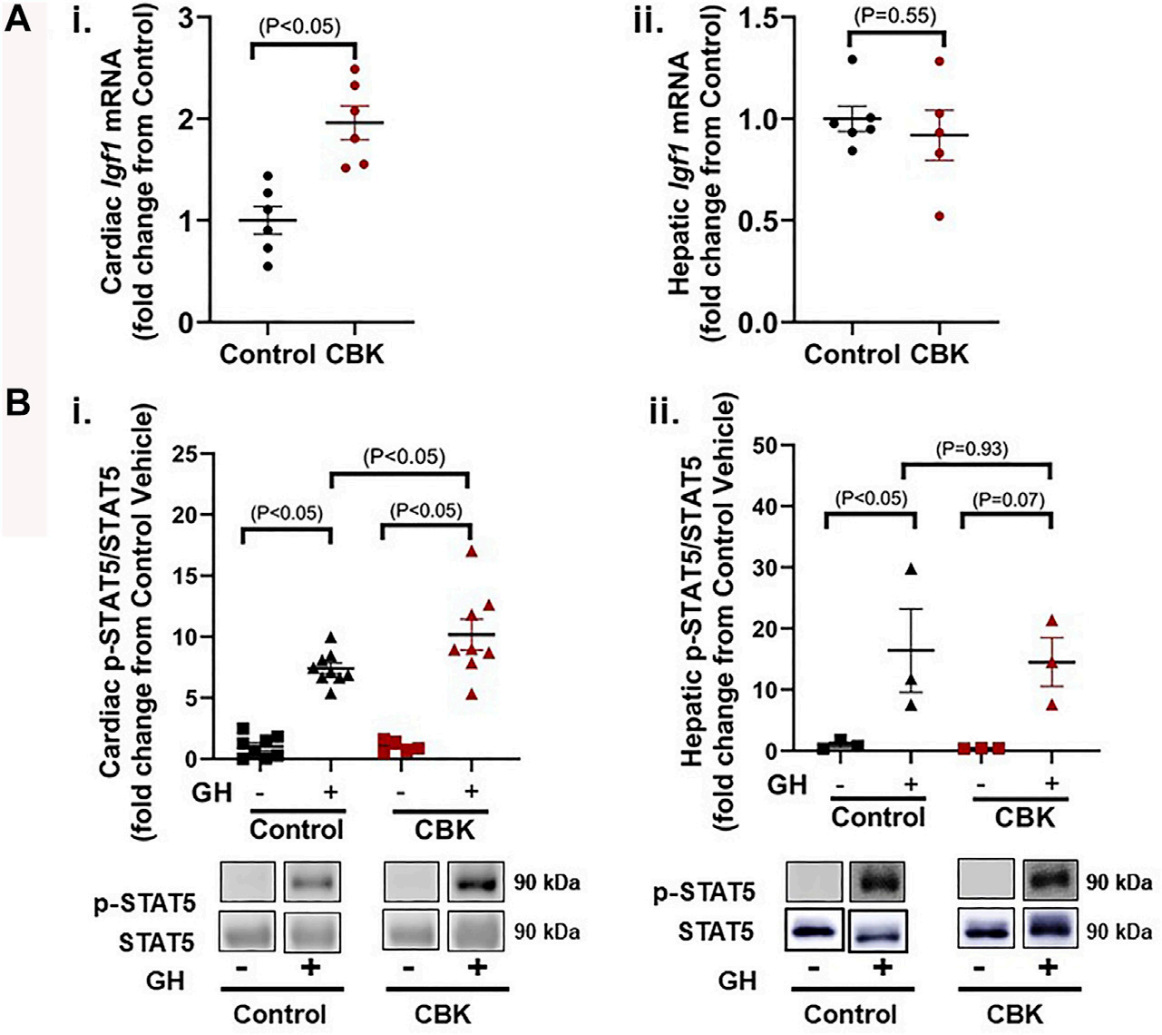
Markers of GH sensitivity in hearts and livers of 16 week old CBK and littermate control mice. **(A)** *Igf1* mRNA levels in hearts (i) and livers (ii) of naive mice (*n* = 5–6). **(B)** p-STAT5/STAT5 ratio in hearts (i; *n* = 5–9) and livers (ii; *n* = 3) 5 min after injection of mice with human recombinant GH (50 μg/kg body weight i.v.) or vehicle (saline). Data/samples were collected at ZT12. All data are reported as mean ± SEM.

The mechanisms by which circadian disruption leads to cardiac hypertrophy remain unknown. A recent unbiased transcriptomic analysis of CBK hearts revealed chronic induction of *Igf1* mRNA (Young et al., 2014); RT-PCR data presented in Figure 2Ai confirm an approximately 2-fold increase in *Igf1* mRNA levels in CBK hearts isolated from 16 week old mice (relative to littermate controls). In contrast, hepatic *Igf1* levels are unaltered in CBK mice (Figure 2Aii). Expression of *Igf1* is regulated by a number of pathways, including growth hormone (GH) signaling (Chia, 2014). This led us to hypothesize that GH signaling was potentially augmented in CBK hearts. To test this hypothesis, 16 week old control and CBK mice were challenged with either GH (50 μg/kg I.V.) or saline (vehicle control) for 5 min, followed by tissue isolation and subsequent assessment of STAT5 phosphorylation. GH administration led to increased cardiac p-STAT5^Tyr694^ levels (relative to vehicle controls), independent of genotype (Figure 2Bi). Importantly, CBK hearts exhibited an augmented increase in p-STAT5 in response to GH administration (relative to littermate controls; Figure 2Bi). In contrast, control and CBK mouse livers exhibited similar increases in p-STAT5 following GH challenge (Figure 2Bii). These data are consistent with the hypothesis that disruption of the cardiomyocyte circadian clock selectively augments cardiac GH sensitivity.

### Growth Hormone Receptor Haploinsufficiency Attenuates the Hypertrophic Phenotype in CBK Mice

**FIGURE 3:**
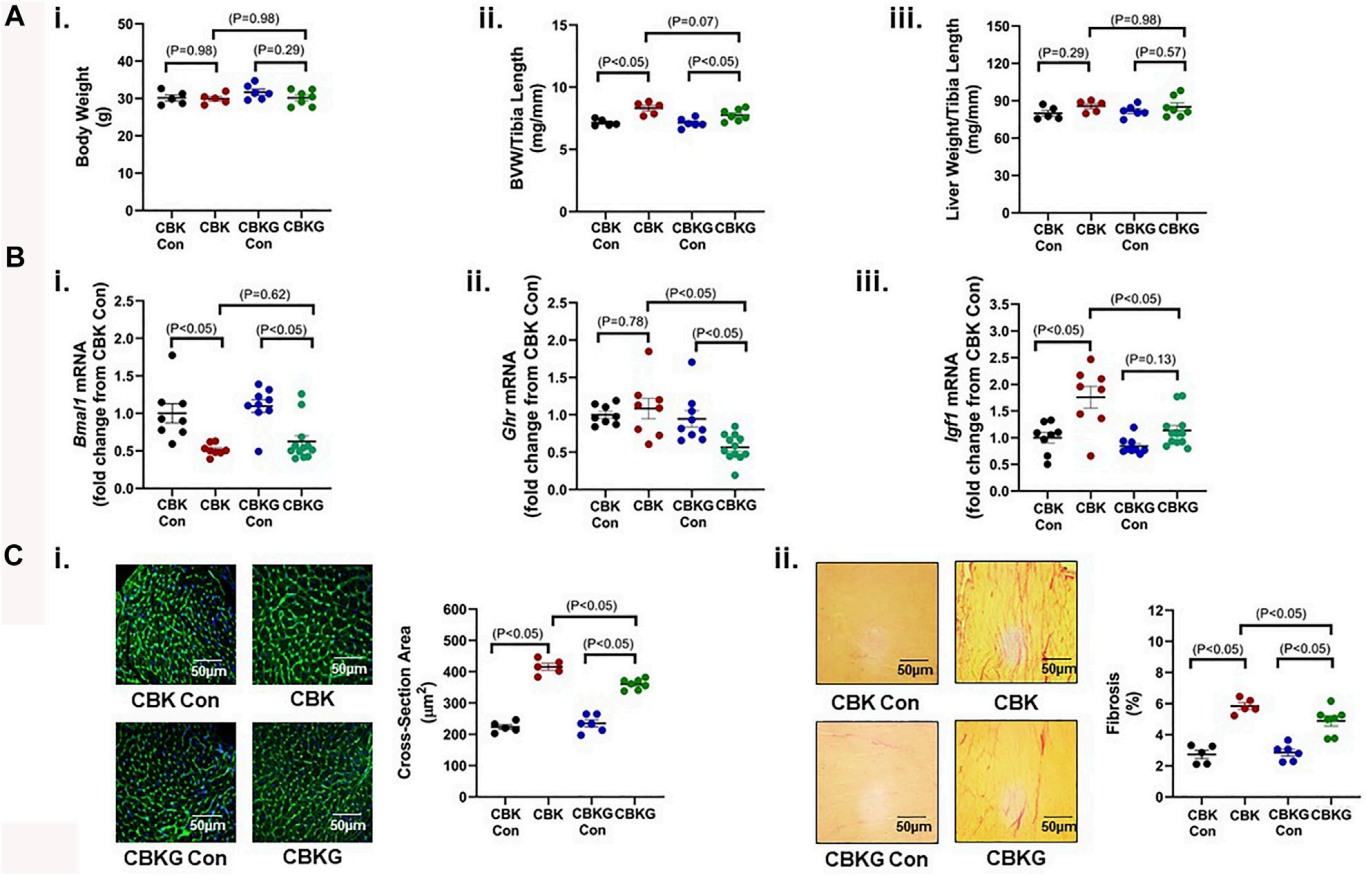
Phenotypic differences between 16 week old CBK, CBKG, and littermate control mice at gravimetric, gene expression, and histologic levels. **(A)** Body weight (i), biventricular weight to tibia length ratio (BVW/TL; ii), and liver weight to tibia length ratio (iii) (*n* = 5–8). **(B)** Cardiac *Bmal1* (i), *Ghr* (ii), *Igf1* (iii), and *Myh7* (iv) mRNA levels (*n* = 6). **(C)** Cardiomyocyte size (i) and interstitial fibrosis (ii) (*n* = 5–8). Data/samples were collected at ZT20. All data are reported as mean ± SEM.

Given that CBK hearts exhibit increased GH sensitivity, we hypothesized that chronic activation of GH/IGF1 signaling in CBK hearts contributes to the cardiac hypertrophic phenotype observed. In order to test this hypothesis, one allele of the GH receptor (GHR) was genetically deleted in CBK mice in a cardiomyocyte-specific manner (termed CBKG); this haploinsufficiency strategy was employed in an attempt to normalize GH/IGF1 signaling in CBK hearts, as opposed to complete inactivation below control levels. 16 week old CBK, CBKG, and littermate control mice were initially investigated at gravimetric, molecular, and histologic levels. At the gravimetric level, CBK mice exhibited increased BVW (16% relative to littermate controls), in the absence of alterations in liver or body weight; although the BVW was also significantly increased in CBKG hearts (9% relative to littermate controls), a trend (*p* = 0.07) was observed for GHR haploinsufficiency to attenuate this parameter (*i.e.,* CBK versus. CBKG; Figure 3A). As predicted, CBK hearts exhibit decreased *Bmal1* mRNA levels, relative to their littermate floxed controls (Figure 3Bi). Consistent with the model, *Ghr* mRNA levels were significantly decreased in CBKG hearts (relative to littermate controls); no difference in *Ghr* mRNA levels were observed between CBK and control hearts (Figure 3Bii). Importantly, elevated *Igf1* mRNA levels observed in CBK hearts were significantly attenuated in CBKG hearts (Figure 3Biii). In contrast, *Bmal1*, *Ghr*, and *Igf1* mRNA levels were equivalent in livers isolated from the 4 experimental groups (Supplementary Figure S1). Importantly, increased cardiomyocyte cross-sectional area and interstitial fibrosis observed in CBK hearts were significantly attenuated by GHR haploinsufficiency (Figure 3C). Collectively, these data are consistent with the hypothesis that chronic activation of GH/IGF1 signaling following genetic disruption of the cardiomyocyte circadian clock contributes to the hypertrophic phenotype.

### Cardiomyocyte-specific Growth Hormone Receptor Haploinsufficiency in CBK Mice Attenuates Age-Onset Adverse Remodeling, Cardiomyopathy, and Mortality

**FIGURE 4:**
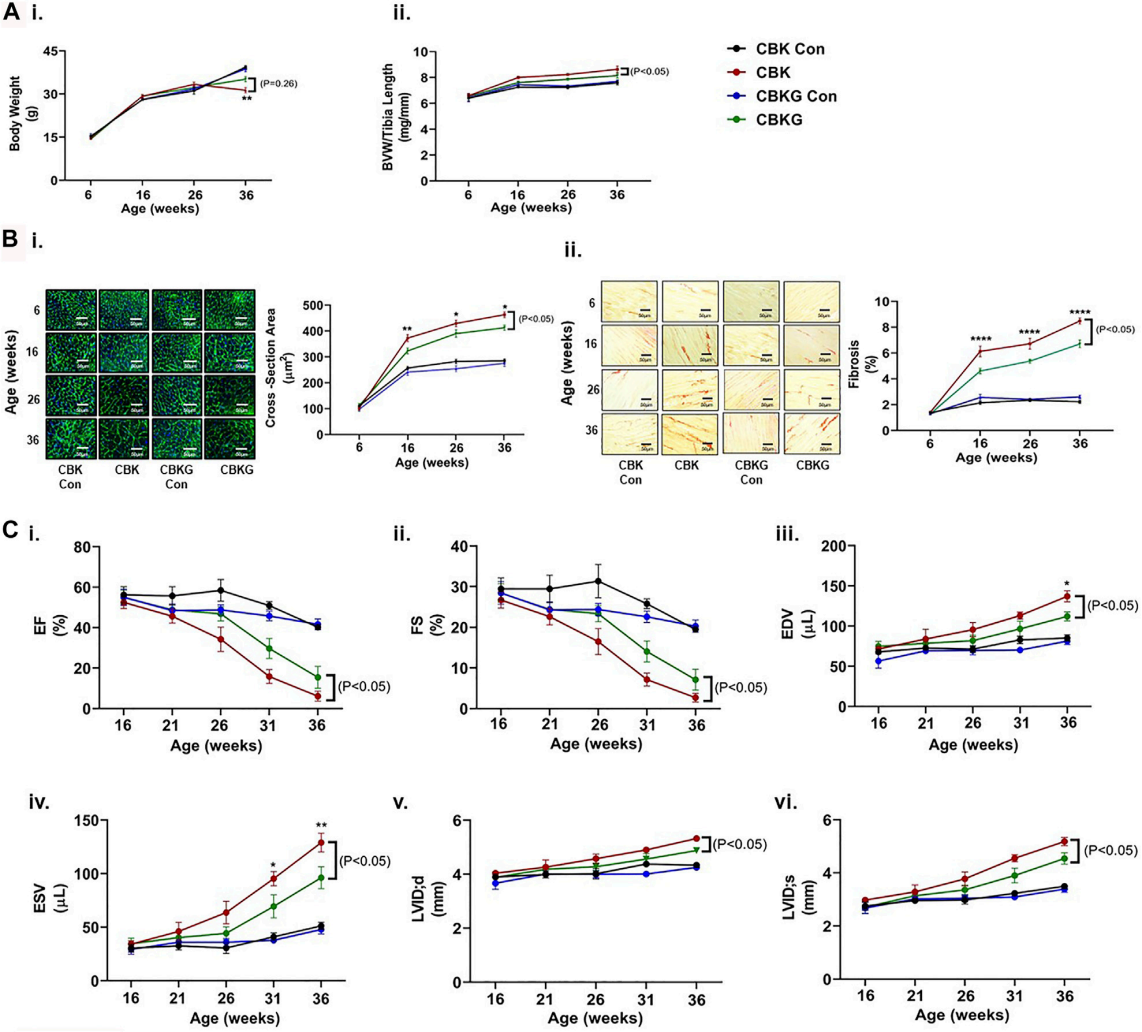
Age-related differences between CBK, CBKG, and littermate control mice at gravimetric, histologic, and functional levels. **(A)** Body weight (i) and biventricular weight to tibia length ratio (BVW/TL; ii) (*n* = 8). **(B)** Cardiomyocyte size (i) and interstitial fibrosis (ii) (*n* = 8. **(C)** Ejection fraction (EF; i), fractional shortening (FS; ii), end diastolic volume (EDV; iii), end systolic volume (ESV; iv), left ventricular inner diameter during diastole (LVIDd; v), left ventricular inner diameter during systole (LVIDs; vi) (*n* = 5–12). Data/samples were collected at ZT12. All data are reported as mean ± SEM. *p < 0.05 for age-matched CBK vs. CBKG mice.

As highlighted above, prior studies report that CBK mice develop age-onset cardiomyopathy and decreased longevity (Young et al., 2014). To determine whether GHR haploinsufficiency attenuates these parameters, CBK and CBKG mice were characterized at gravimetric, histologic, and functional levels, at multiple ages (up to 36 weeks old). At baseline (6 weeks of age), no significant differences were observed for either body weight or BVW between the 4 experimental groups (Figure 4Ai-ii). Both body weight and BVW increased as a function of age, independent of genotype (*i.e.,* age main effect; Figure 4Ai-ii and Supplementary Table S1). Consistent with prior reports, BVW increased in CBK mice to a greater extent (relative to littermate controls; Figure 4Aii and Supplementary Table S1). Similarly, CBKG mice exhibited increased BVW relative to littermate controls (Figure 4Aii and Supplementary Table S1). However, BVW was significantly lower in CBKG mice relative to CBK mice (Figure 4Aii and Supplementary Table S1). Essentially identical patterns were seen at the histologic level; age-associated changes observed for CBK hearts for both cardiomyocyte cross sectional area and interstitial fibrosis were significantly attenuated in CBKG hearts, in the absence of differences at baseline (6 weeks of age; Figure 4Bi-ii and Supplementary Table S1). Next, serial echocardiography was employed to non-invasively assess age-onset alterations in contractile function. At baseline (16 weeks of age for functional parameters), no significant differences in echocardiographic parameters were observed between the 4 experimental groups (Figure 4Ci–iv and Supplementary Table S2). Both CBK and CBKG mice exhibited an age-dependent decline in ejection fraction, associated with increased end diastolic/systolic volumes and left ventricular inner diameter (during systole; Figure 4Ci–iv and Supplementary Tables S1, S2). However, the extent of these age-dependent changes in contractile parameters was significantly attenuated in CBKG mice (relative to CBK mice; Figure 4Ci–iv and Supplementary Tables S1, S2). Consistent with previously published studies (Young et al., 2014), CBK mice exhibited a significant reduction in survival relative to littermate controls (67% mortality at 36 weeks of age; *p* < 0.05); in contrast, CBKG mice did not exhibit a significant reduction in survival relative to littermate controls. Collectively, these data suggest that excessive GH/IGF1 signaling in the heart contributes towards age-onset cardiomyopathy following disruption of the cardiomyocyte circadian clock.

## Discussion

Growth hormone (GH) exerts pleotropic actions in peripheral tissues, including striking effects on protein synthesis and metabolism (Møller and Jørgensen, 2009). Following secretion from the anterior pituitary, GH binds to the cell-surface GH receptor (GHR), causing the activation of associated Janus kinase (JAK) 2 (Argetsinger et al., 1993; Frank and Messina, 2002). JAK2 activates a number of downstream signaling molecules including STAT5 (Argetsinger and Carter-Su, 1996). Upon phosphorylation, STAT5 forms a homodimer and translocates to the nucleus, where it regulates expression of a number of genes, including *Igf1* (Chia, 2014). Once secreted from the cell, IGF1 elicits a range of biological effects in paracrine, autocrine, and endocrine fashion, *via* binding to the cell-surface IGF1 receptor (IGF-1R) (Favelyukis et al., 2001). In numerous tissues, IGF1 signaling promotes anabolic processes through the combined actions of MAP kinases (*e.g.,* ERK1) and the Akt/mTOR/4-EBP1 signaling axis (Troncoso et al., 2014). Cardiac IGF1 signaling is typically considered physiologic in nature, being important for the normal growth of the heart during development and following exercise training (Kim et al., 2008). However, chronic activation of this signaling axis leads to pathologic hypertrophy of the heart, contractile dysfunction, and heart failure. For example, overexpression of human IGF1 in skeletal and cardiac muscle of the mouse results in cardiac hypertrophy, which transitions to contractile dysfunction with age (Delaughter et al., 1999). Individuals with acromegaly, characterized by high circulating levels of GH and IGF1, also display concentric bi-ventricular hypertrophy with ensuing diastolic and systolic cardiac dysfunction (Clayton, 2003; Melmed, 2009; Arcopinto et al., 2013). Additionally, other studies have linked increased levels of GH and IGF1 with cardiomegaly and hypertrophy of cardiomyocytes (Donohue et al., 1994; Cittadini et al., 1996; Reiss et al., 1996). Moreover, circulating GH levels post-AMI (acute myocardial infarction) are prognostic for subsequent major adverse cardiac events (Ng et al., 2015). Collectively, these observations suggest that excess GH/IGF-1 signaling results in hypertrophic cardiomyopathy and increased mortality.

We have previously shown that GH promotes protein synthesis in isolated cardiomyocytes (Lu et al., 2001). This process exhibits time-of-day-dependent fluctuations in the heart, peaking at the beginning of the sleep phase (McGinnis et al., 2017). Unlike other factors that promote protein synthesis (*e.g.,* insulin, amino acids, sheer stress), GH levels are elevated during the sleep phase; more accurately, growth hormone is secreted in a pulsatile fashion, and the amplitude of these pulses are increased during the sleep phase (Goji, 1993; Jaffe et al., 1998; Avram et al., 2005). Such observations suggest that GH may contribute towards augmented cardiac protein synthesis during the sleep phase. Interestingly, time-of-day rhythms in cardiac protein synthesis also appear to be dependent on the cardiomyocyte circadian clock, as genetic disruption of this intrinsic timekeeping mechanism (i.e., cardiomyocyte-specific BMAL1 knockout [CBK]) abolishes daily rhythms in cardiac protein synthesis (McGinnis et al., 2017). Moreover, hearts of CBK mice exhibit chronically elevated rates of protein synthesis, associated with increased activation of the Akt/mTOR/4-EBP1 signaling axis, hypertrophy, and age-dependent contractile dysfunction (Young et al., 2014; Ingle et al., 2015; McGinnis et al., 2017). This phenotype of CBK hearts mirrors that of mice overexpressing human IGF1 (Delaughter et al., 1999). Such observations led us to hypothesize that augmented GH/IGF1 signaling in the heart following genetic disruption of the cardiomyocyte circadian clock may contribute towards adverse cardiac remodeling.

Here, we report that CBK hearts exhibit increased sensitivity to GH. More specifically, exogenous GH elicits an augmented phosphorylation of STAT5 in hearts of CBK mice, relative to littermate controls (Figure 2B). Moreover, cardiac *Igf1* mRNA levels are approximately 2-fold higher in CBK mice (compared to littermate controls; Figure 2A), associated with increased activation of ERK and mTOR/4-EBP1 signaling, as well as adverse remodeling (cardiomyocyte hypertrophy and interstitial fibrosis; Figure 1). In an attempt to determine the contribution of augmented GH/IGF1 signaling in CBK hearts towards adverse cardiac remodeling, we next employed a genetic strategy to normalize cardiac GH sensitivity (through generation of CBK mice with cardiomyocyte GHR haploinsufficiency; i.e., CBKG mice). Consistent with the model, cardiac *Igf1* mRNA levels were essentially equivalent between CBKG and control mice (Figure 3). Importantly, age-onset adverse remodeling and contractile dysfunction were significantly attenuated in CBKG mice (relative to CBK mice; Figure 4). Collectively, these observations are consistent with the hypothesis that augmented GH signaling in CBK hearts, possibly *via* enhanced local IGF1 action, contributes towards cardiomyopathy development.

Although the present study has numerous strengths, a number of notable limitations also exist. The first is a lack of establishment of the mechanistic links between cardiomyocyte BMAL1 deletion and augmented cardiac GH sensitivity. Given the transcriptional nature of the circadian clock mechanism, we initially screened a number of critical components in the GH signaling pathway at the mRNA level. However, no significant differences in *Ghr*, *Jak2*, or *Socs2* were observed between CBK and littermate control hearts (Supplementary Figure S2). Interestingly, a recent study by Lyu *et al* reported that GH responsiveness was decreased in liver and skeletal muscle of germline BMAL1 knockout mice, which was associated with increased levels of SOC3 and PTP1B (established antagonists of GH signaling) (Lyu et al., 2021). The reasons for these opposing results are unclear, but may relate to differences in the models (i.e., germline versus cell-type specific BMAL1 deletion) and/or tissues (i.e., liver and skeletal muscle versus heart) investigated. In the case of model differences, germline BMAL1 deletion is associated with profound neurohumoral and behavioral alterations (leading to cardiometabolic disease), which are not observed in CBK mice (Turek et al., 2005; Marcheva et al., 2010; Young et al., 2014); neurohumoral factors (e.g., FGF21), behaviors (e.g., feeding/fasting), and cardiometabolic diseases (e.g., obesity) are known to affect GH responsiveness (Beauloye et al., 2002; Inagaki et al., 2008). With regards to potential tissue-specificity, numerous examples exist wherein the heart responds to stimuli/stresses in a manner that is distinct relative to other tissues. Protein and glycogen synthesis serve as good examples, as both are influenced by GH signaling. During fasting, protein and glycogen synthesis decrease in skeletal muscle and the liver; in contrast, fasting either has no effect or increases these metabolic processes in the heart. Second, another unanswered question relates to whether the observed augmentation of cardiac GH sensitivity in CBK mice is model-specific, or whether it is more generalizable to circadian disruption. We have previously reported that simulated shiftwork in mice (through manipulation of the light/dark cycle) results in cardiomyocyte hypertrophy and interstitial fibrosis (Durgan et al., 2011a). Investigating *Igf1* mRNA as an indirect marker of GH action in these same heart samples revealed increased cardiac *Igf1* mRNA following simulated shiftwork (Supplementary Figure S3), suggesting that environment-mediated circadian disruption may augment cardiac GH sensitivity. Finally, it is noteworthy that although GHR haploinsufficiency normalized cardiac *Igf1* mRNA in CBKG hearts, the current study did not investigate GH signaling. Moreover, adverse cardiac remodeling and cardiomyopathy development was only partially attenuated, indicating that GH/IGF1-independent mechanisms also contribute towards cardiac pathology in CBK mice.

In summary, the current study reveals that cardiomyocyte-specific deletion of BMAL1 increases sensitivity of the heart to GH. This is associated with a chronic increase in cardiac GH/IGF signaling and adverse cardiac remodeling. Normalization of the cardiac GH/IGF1 axis through genetic means partially attenuates age-onset cardiomyopathy. These data may provide insight regarding the mechanisms linking circadian disruption with increased heart disease risk.

## Data Availability

The original contributions presented in the study are included in the article/Supplementary Material, further inquiries can be directed to the corresponding authors.

## Supplementary Material

Supplementary Figure S1 Hepatic gene expression in 16 week old CBK, CBKG, and littermate control mice. Hepatic *Bmal1* (i), *Ghr* (ii), and *Igf1* (iii) mRNA levels (*n*=6). Samples were collected at ZT20. All data are reported as mean ± SEM.

Supplementary Figure S2 Cardiac gene expression in 16 week old CBK and littermate control mice. Cardiac *Ghr* (i), *Jak2* (ii), and *Socs2* (ii) mRNA levels (*n*=6). Samples were collected at ZT20. All data are reported as mean ± SEM.

Supplementary Figure S3 Effects of simulated shift work (SSW) on cardiac Igf1 mRNA levels. Mice were subjected to a SSW protocol, as described previously (Durgan DJ, Tsai JY, Grenett MH, Pat BM, Ratcliffe WF, Villegas-Montoya C, Garvey ME, Nagendran J, Dyck JR, Bray MS, Gamble KL, Gimble JM, and Young ME. Evidence suggesting that the cardiomyocyte circadian clock modulates responsiveness of the heart to hypertrophic stimuli in mice. *Chronobiol Int* 28: 187-203, 2011). Briefly, mice were subjected to a biweekly 12-h phase shift in the light/dark cycle for a total of 16 weeks; control mice were maintained in a standard 12-h light and 12-h dark cycle. Samples were collected at ZT6. All data are reported as mean ± SEM (*n*=5–7).

Supplementary Figure S4 Original Western Blot images utilized for data presented. In order to minimize the contribution that position on the gel might have on outcomes, samples were randomized on gels; samples were re-ordered post-imaging, only for the sake of illustration of representative images in the figures. Original, uncropped images are presented here for Figure 1C.

Supplementary Figure S5 Original Western Blot images utilized for data presented. In order to minimize the contribution that position on the gel might have on outcomes, samples were randomized on gels; samples were re-ordered post-imaging, only for the sake of illustration of representative images in the figures. Original, uncropped images are presented here for Figure 2B. OEG represents “Other Experimental Group” (not reported).

Supplementary Table S1 Two-way ANOVA for data in Figure 4. BVW/TL, biventricular weight to tibia length ratio; EF, ejection fraction; EDV, end-diastolic volume; ESV, end-systolic volume; LVID; s, left ventricular inner diameter during systole.

Supplementary Table S2 Echocardiographic parameters in 36 week old CBK, CBKG, and littermate control mice. IVS; d, inner ventricular septal wall thickness during diastole; LVID; d, left ventricular inner diameter during diastole; LVPW; d, left ventricular posterior wall thickness during diastole; IVS; s, inner ventricular septal wall thickness during systole; LVID; s, left ventricular inner diameter during systole; LVPW; s, left ventricular posterior wall thickness during systole; HR, heart rate; V; s, left ventricular volume during systole; V; d, left ventricular volume during diastole; SV, stroke volume; EF, ejection fraction; FS, fractional shortening; CO, cardiac output. $*p* < 0.05 for CBK Con versus CBK; #*p* < 0.05 for CBKG Con versus CBKG; **p* < 0.05 for CBK versus CBKG mice.
