# Supplementary figures and images for: Augmented Cardiac Growth Hormone Signaling Contributes to Cardiomyopathy Following Genetic Disruption of the Cardiomyocyte Circadian Clock
Source: Front Pharmacol. 2022 Feb 16;13:836725. doi: 10.3389/fphar.2022.836725 (PMC8888912; doi:10.3389/fphar.2022.836725)

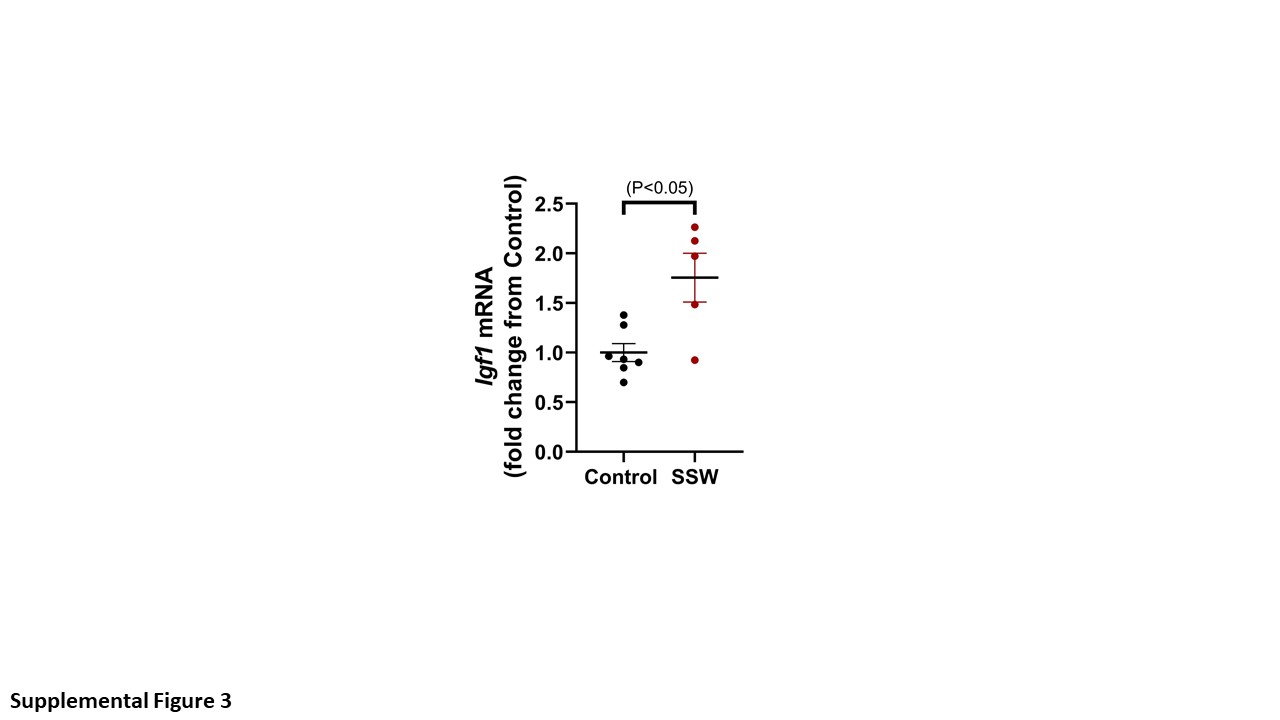

Supplement: Supplementary file 1 [file Image3.JPEG]

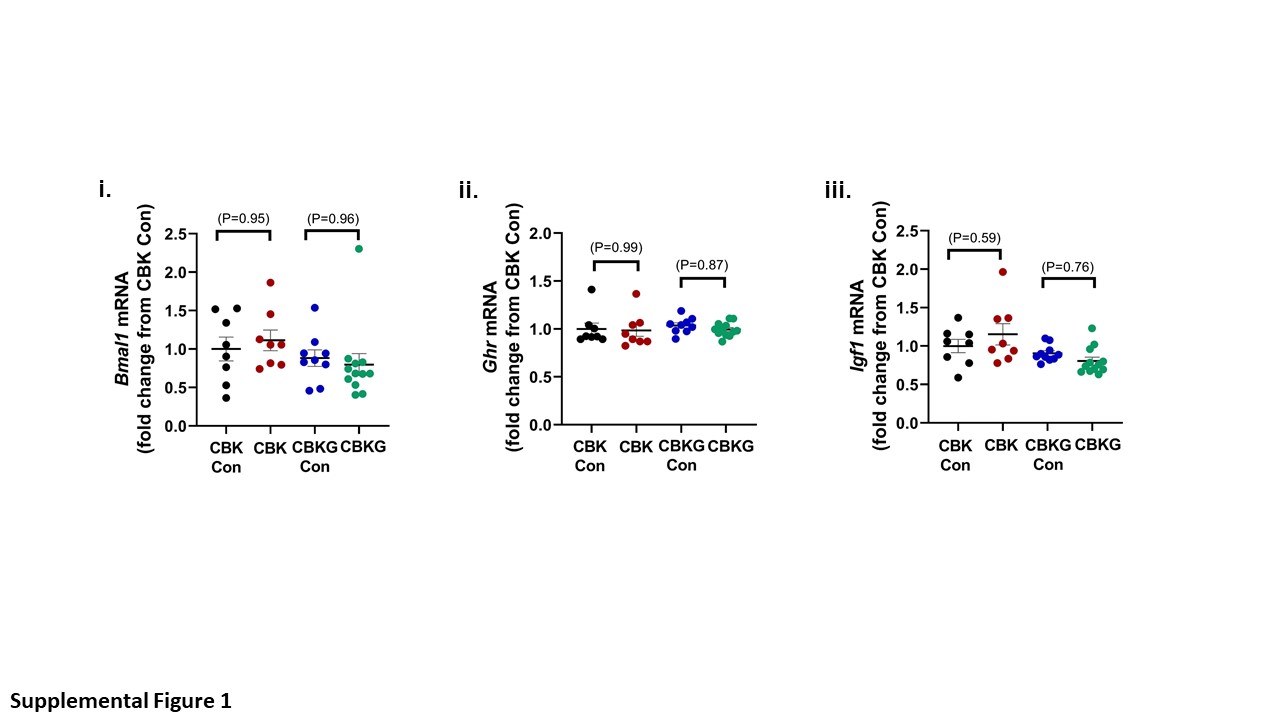

Supplement: Supplementary file 3 [file Image1.JPEG]

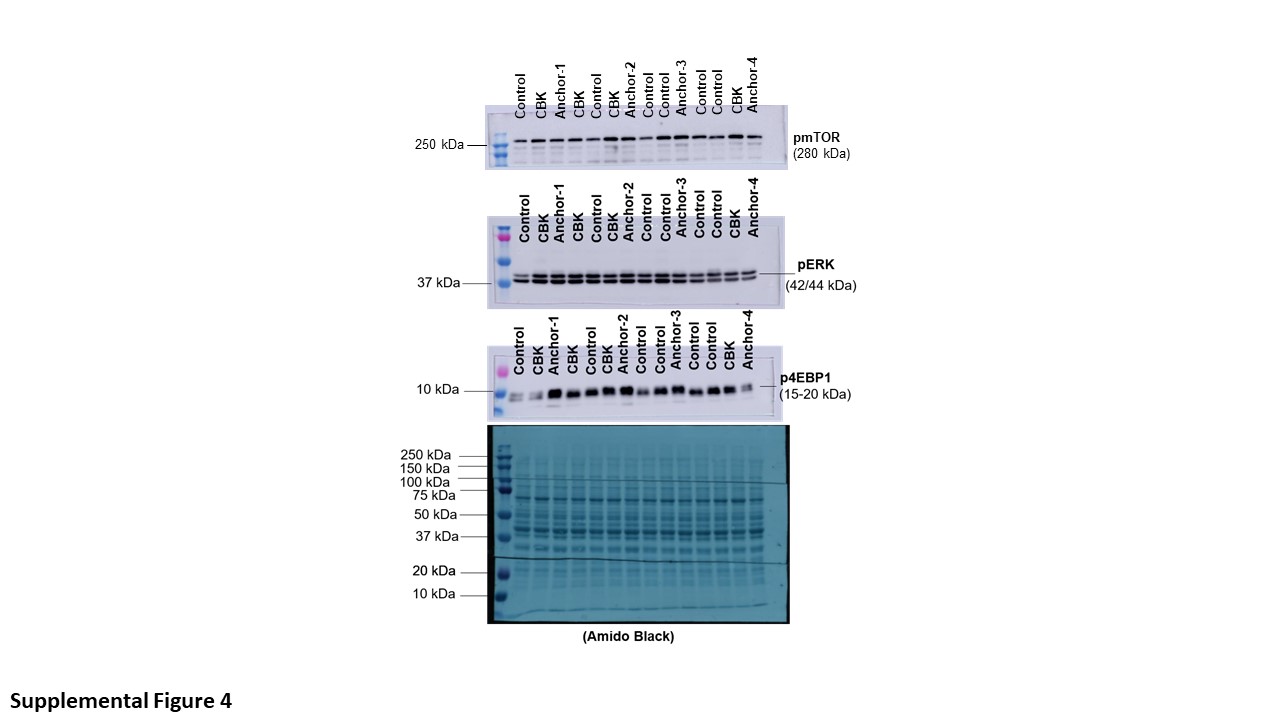

Supplement: Supplementary file 4 [file Image4.JPEG]

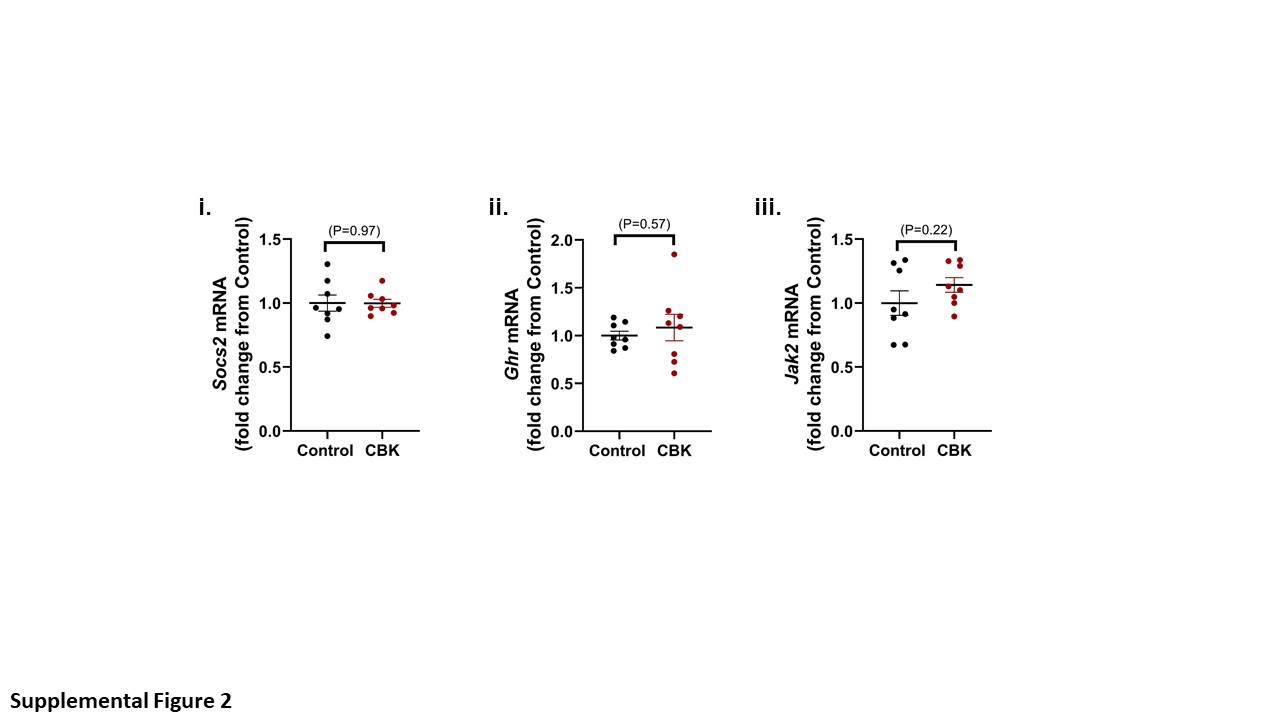

Supplement: Supplementary file 5 [file Image2.JPEG]

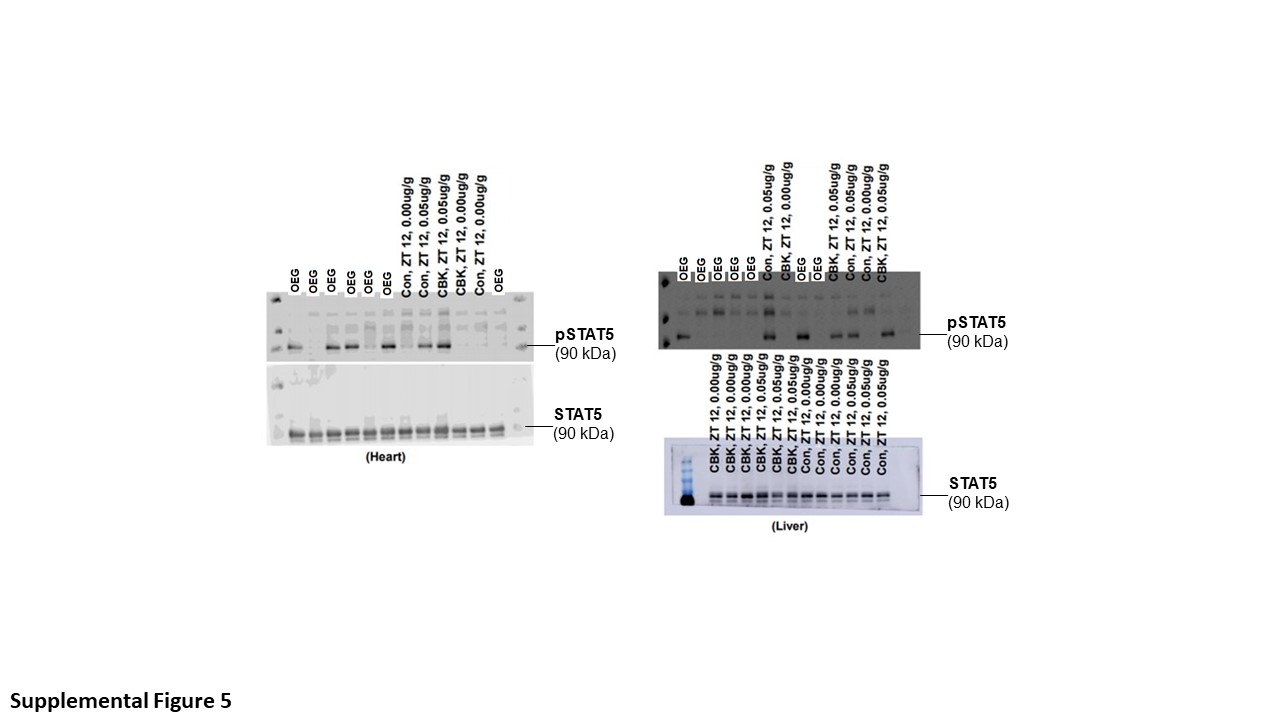

Supplement: Supplementary file 6 [file Image5.JPEG]
