# Supplementary material for: Augmented Cardiac Growth Hormone Signaling Contributes to Cardiomyopathy Following Genetic Disruption of the Cardiomyocyte Circadian Clock
Source: Front Pharmacol. 2022 Feb 16;13:836725. doi: 10.3389/fphar.2022.836725 (PMC8888912; doi:10.3389/fphar.2022.836725)
Supplement: Supplementary file 2 [file Table1.DOCX]

|  | **Main effect between the groups (P-Values)** | | | |
| --- | --- | --- | --- | --- |
|  | **CBK Con**  **Versus**  **CBK** | **CBKG Con Versus CBKG** | **CBK Con**  **Versus CBKG Con** | **CBKG**  **Versus**  **CBK** |
| **Body weight (Fig 4Ai)** | P<0.05 | P=0.17 | P=0.99 | P=0.26 |
| **BVW/TL (Fig 4Ai)** | P<0.05 | P<0.05 | P=0.34 | P<0.05 |
| **Cardiomyocytes Cross-Section Area (Fig 4Bi)** | P<0.05 | P<0.05 | P<0.05 | P<0.05 |
| **Interstitial Fibrosis (Fig 4Bii)** | P<0.05 | P<0.05 | P<0.05 | P<0.05 |
| **EF (Fig 4Ci)** | P<0.05 | P<0.05 | P<0.05 | P<0.05 |
| **EDV (Fig 4Cii)** | P<0.05 | P<0.05 | P<0.05 | P<0.05 |
| **ESV (Fig 4Ciii)** | P<0.05 | P<0.05 | P=0.92 | P<0.05 |
| **LVID;s (Fig 4Civ)** | P<0.05 | P<0.05 | P=0.65 | P<0.05 |

**Supplemental Table 1. Two-way ANOVA for data in Figure 4.** BVW/TL, biventricular weight to tibia length ratio; EF, ejection fraction; EDV, end-diastolic volume; ESV, end-systolic volume; LVID;s, left ventricular inner diameter during systole.

| **Parameters** | **Unit** | **CBK Con**  **(n=12)** | **CBK**  **(n=5)** | **CBKG Con**  **(n=11)** | **CBKG**  **(n=7)** |
| --- | --- | --- | --- | --- | --- |
| **IVS;d** | mm | 0.90 ± 0.07 | 0.86 ± 0.08 | 0.99 ± 0.05 | 0.92 ± 0.08 |
| **LVID;d** | mm | 4.33 ± 0.09 | 5.32 ± 0.11^$^ | 4.25 ± 0.09 | 4.87 ± 0.10^#, *^ |
| **LVPW;d** | mm | 0.72 ± 0.03 | 0.80 ± 0.03 | 0.72 ± 0.03 | 0.75 ± 0.02 |
| **IVS;s** | mm | 1.14 ± 0.08 | 0.91 ± 0.09 | 1.27 ± 0.06 | 1.03 ± 0.06 |
| **LVID;s** | mm | 3.49 ± 0.10 | 5.18 ± 0.15^$^ | 3.39 ± 0.12 | 4.54 ± 0.21^#, *^ |
| **LVPW;s** | mm | 0.98 ± 0.04 | 0.82 ± 0.02^$^ | 0.99 ± 0.05 | 0.81 ± 0.03^#^ |
| **HR** | BPM | 467.71 ± 8.64 | 441.00 ± 64.69 | 447.00 ± 12.13 | 490.14 ± 26.33 |
| **V;s** | uL | 51.12 ± 3.43 | 128.88 ± 8.84^$^ | 47.77 ± 4.21 | 96.13 ± 10.34^#, *^ |
| **V;d** | uL | 85.15 ± 4.09 | 137.00 ± 6.89^$^ | 81.17 ± 4.05 | 112 ± 5.75^#, *^ |
| **SV** | uL | 34.02 ± 0.95 | 8.11 ± 3.06^$^ | 33.39 ± 1.97 | 15.87 ± 5.05^#, *^ |
| **EF** | % | 40.25 ± 1.43 | 6.08 ± 2.45^$^ | 41.62 ± 2.69 | 15.41 ± 5.39^#, *^ |
| **FS** | % | 19.46 ± 0.78 | 2.69 ± 1.08^$^ | 20.28 ± 1.49 | 7.11 ± 2.58^#, *^ |
| **CO** | mL/min | 12.78 ± 0.71 | 6.67 ± 1.04^$^ | 11.77 ± 1.06 | 9.12 ± 1.32 |

**Supplemental Table 2. Echocardiographic parameters in 36 week old CBK, CBKG, and littermate control mice.** IVS;d, inner ventricular septal wall thickness during diastole; LVID;d, left ventricular inner diameter during diastole; LVPW;d, left ventricular posterior wall thickness during diastole; IVS;s, inner ventricular septal wall thickness during systole; LVID;s, left ventricular inner diameter during systole; LVPW;s, left ventricular posterior wall thickness during systole; HR, heart rate; V;s, left ventricular volume during systole; V;d, left ventricular volume during diastole; SV, stroke volume; EF, ejection fraction; FS, fractional shortening; CO, cardiac output. $, p<0.05 for CBK Con versus CBK; #, p<0.05 for CBKG Con versus CBKG; *, p<0.05 for CBK versus CBKG mice.
